# A Case—Amalgam

**Published:** 1883-10

**Authors:** 


### A Case—Amalgam.

Miss W. presented a few weeks ago a right superior third molar, on the buccal surface of which was a large amalgam filling, extending almost across the side of the tooth, and about a line beneath the margin of the gum. This filling had been inserted about two years before this examination.

There was no sensitiveness of the tooth, either from pulp or inflamed dentine at the time the amalgam was introduced. There had been from the time the filling was introduced, frequent paroxysms of pain in the parts about the tooth which seemed to extend from it.

It was suggested by a dentist and physician that it was neuralgia, and did not proceed from the tooth, inasmuch as there was no soreness about the tooth, the gum showing only a little irritation of its margin, where it was in contact with the filling.

The pain was so frequent and so severe that the lady thought of having the tooth extracted.

But upon examination, it was proposed to remove the filling, ascertain the condition of the cavity, and await the result. Upon the removal of the filling there was no exposure of the pulp nor sensitiveness of the dentine, a jet of cold water produced no pain, nor was there any decay in the cavity. A pledget of cotton, moistened with diluted creosote was packed into the cavity, and the patient dismissed for about a week; during this time not the slightest pain or discomfort was experienced by the patient. At the end of this time the margin of the gum was less irritated, and was somewhat pressed out of the way, an oxyphosphate filling was introduced. Since that time, now about two months, not a tinge of pain, nor soreness has he experienced in, nor about the tooth.

It should then be said that the amalgam filling was a good one, so far as an amalgam filling can be good, it was well inserted, well finished and had no decay about it.

I have known many such cases in connection with amalgam fillings, but not a case of this sort with any other kind of filling.

